# The association between telework and household food insufficiency in the United States

**DOI:** 10.1017/S1368980026103085

**Published:** 2026-07-13

**Authors:** Andrea Leschewski, Bridget Yeboah Bafowaa, Triwit Ariyathugun

**Affiliations:** 1 Ness School of Management & Economics, South Dakota State University, USA; 2 Agricultural Economics & Rural Sociology, Auburn University, USA; 3 Independent researcher

**Keywords:** Food insufficiency, Food hardship, Telework, Work from home, Remote work

## Abstract

**Objective:**

To analyse the association between telework and household food insufficiency in the United States.

**Design:**

Cross-sectional study. Probit regression models were used to examine the association between telework (having at least one teleworking household member) and food insufficiency. Sensitivity analyses were conducted to examine the relationship across income levels and time periods.

**Setting:**

United States Census Bureau’s Household Pulse Survey, Phases 3·1 to 3·10 (April 2021 to October 2023).

**Participants:**

326 876 households with income below 200 % of the federal poverty line (FPL).

**Results:**

Telework was associated with a 4·4 percentage point decrease in the probability of food insufficiency among households with low income (predicted probabilities: 20·3 % (telework) *v*. 24·5 % (no telework); *P* < 0·01). The inverse relationship between telework and food insufficiency held across income levels (< 100 % FPL and 100–200 % FPL) and time periods (pandemic and post-pandemic).

**Conclusions:**

Telework is associated with reduced household food insufficiency in the United States. Policies and interventions aimed at promoting telework adoption, and equitable telework opportunities may serve as valuable tools in addressing food hardship and advancing public health goals.

The COVID-19 pandemic accelerated telework adoption. As shown in Figure 1, the percentage of the United States workforce engaging in telework increased from 24 % in 2019 to over 38 % in 2021^(1)^. Telework adoption remains above pre-pandemic levels, with 33 % of workers reporting telework in 2024^(1)^. Telework is defined as an arrangement where employees work from an alternative site for a set number of days each pay period^(2)^. While definitions vary, telework is often used interchangeably with the terms ‘remote work’ and ‘working from home’^(3)^.

**Figure 1.**
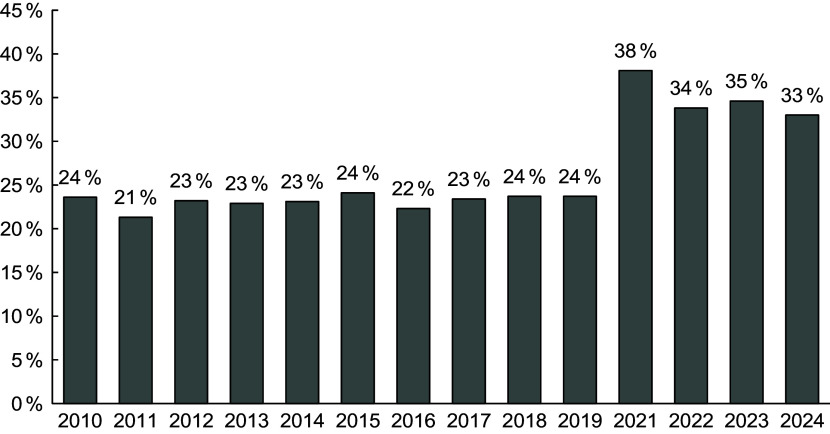
Percent of employed persons in the United States who teleworked on an average day (2010–2024). Source: American Time Use Survey (ATUS). Note: ATUS was not published in 2020 due to a pause in data collection.

Alternative work arrangements, such as telework, can have significant implications for public health. Notably, the literature identifies work arrangements as a determinant of food insecurity and food insufficiency^(4–6)^. Defined as limited or uncertain access to sufficient, nutritious food for an active, healthy life, food insecurity is a major public health challenge associated with decreased nutrient intake and increased incidence of chronic diseases and mental health problems^(7–9)^. Food insufficiency is a more severe form of food hardship where households sometimes or often do not have enough to eat^(10)^. Annual medical costs attributable to food hardship totalled nearly $52 billion in 2016^(11)^. Households with members working in part-time positions, with variable hours or working multiple jobs are more likely to experience food hardship than those with full-time employment^(4,5)^. Long hours and variable shifts are further linked to a higher likelihood of skipping meals^(12)^. These connections stem from the instability inherent in nonstandard work arrangements, which often result in unpredictable income and complex household schedules that can hinder consistent access to affordable, nutritious food^(4,13–15)^.

Telework has the potential to affect food hardship through several mechanisms. A comprehensive study on the impacts of telework commissioned by the European Parliament found that teleworking affects household income, time constraints and social isolation^(3)^. Telework adoption often reduces or eliminates the transportation costs associated with commuting^(3)^. The literature indicates reduced transportation costs increase household disposable income, resulting in reduced household food insufficiency^(6,16)^. Reduced commuting time due to telework may further alleviate food insufficiency, particularly for time-constrained households who often opt for more expensive prepared meals to save time^(17)^. By reallocating saved commuting time to home meal preparation – a trend seen during the pandemic – households can acquire a greater quantity of food with the same food budget^(17–19)^.

While telework offers potential benefits for some, it may exacerbate food hardship for the most vulnerable households. Increased expenses associated with teleworking, such as higher utility bills and the need for home office supplies, can strain household budgets and reduce disposable income^(20,21)^. This reduction in household income can lead to increased food hardship as households have less money available for groceries^(16)^. Telework adoption is also associated with social isolation, which can disrupt access to informal food support systems like shared meals with coworkers or employer-provided food. This loss can exacerbate food hardship, as social support networks often serve as a coping strategy for households facing food hardship^(22,23)^.

The existing literature on telework adoption and food hardship is limited. Drawing on data for the general population of US households over a 30-day period from mid-November to mid-December 2020, Coleman-Jensen *et al.* report food insecurity was 3·5 percentage points lower among households with a reference person who was able to telework than the national average^(24)^. In a 2022 study using data from the US Census Bureau’s Household Pulse Survey (HPS), Nelson *et al.* report 59 %, 46 %, 24 % and 14 % of food secure, marginally food secure, low food secure and very low food secure Massachusetts households, respectively, had at least one teleworking adult^(25)^. While these descriptive statistics provide initial insights, they do not account for the underlying sociodemographic factors that affect both telework adoption and food hardship. This study builds on this work in two key ways. First, it focuses on food insufficiency, a more severe measure of food hardship. Second, this study uses multivariate probit regression analysis to move beyond descriptive associations to analyse the association between telework and food insufficiency while controlling for key sociodemographic factors such as income and household composition.

This study contributes to the literature by applying a multivariate framework to analyse the association between telework and food insufficiency in the United States. Leveraging data from the HPS, this study examines the telework-food insufficiency association specifically among households with income below 200 % of the federal poverty line (FPL)^(26)^. Sensitivity analyses are conducted to assess whether there is heterogeneity in the association between telework and food insufficiency by income (< 100 % FPL and 100–200 % FPL) and time period (pandemic and post-pandemic)^(27)^. This study provides initial insight on the implications of the United States’ shift toward telework for food hardship and will enhance understanding of the broader role of work arrangements in addressing public health challenges. These findings bring a public health perspective to ongoing federal telework policy debates, further informing policy decisions regarding regulation of flexible work arrangements as well as policies aimed at addressing spatial and social inequalities that hinder equitable telework adoption.

## Methods

### Household pulse survey overview

The HPS is a publicly available, cross-sectional dataset of US households. Designed and conducted by the US Census Bureau in collaboration with twenty other federal agencies, the survey measures how social and economic issues impacted households in the USA during the COVID-19 pandemic and post-pandemic period. The survey uses a phased approach, in which data were collected and released in phases consisting of several survey weeks. This study uses data from Phases 3·1 to 3·10 (April 2021 to October 2023), as this period was the first to include a measure of telework and preceded significant changes to the survey design in Phase 4. The final study sample includes 326 876 US households with income below 200 % of the FPL.

### Measures

The main outcome of interest in this study is food insufficiency. The HPS measures food insufficiency through the following question: ‘In the last 7 d, which of these statements best describes the food eaten in your household? Select only one answer.’ Possible responses included: (1) ‘Enough of the kinds of food (I/we) wanted to eat’, (2) ‘Enough, but not always the kinds of food (I/we) wanted to eat’, (3) ‘Sometimes not enough to eat’, and (4) ‘Often not enough to eat’. Based on the USDA’s definition of food insufficiency, this study creates a binary measure of food insufficiency, assigning a value of 1 to households selecting responses 3 or 4 (‘sometimes’ or ‘often not enough to eat’) and 0 otherwise^(10)^.

A binary indicator was also created for the main independent variable of interest, telework. A question on telework was added to the HPS in Phase 3·1, with subsequent changes made to the question in Phases 3·2 and 3·4. Table 1 provides a summary of each of the three versions of the telework question, the corresponding response options and how responses for each question translate to values for the telework variable. Despite variation in wording and response options, all three versions of the question ask whether a member of the respondent’s household teleworked in the past week. A binary telework measure was created, with a value of 1 indicating that a household member teleworked at any time during the previous week and 0 otherwise.

**Table 1.** Summary of HPS questions and coding for telework: United States, April 2021 to October 2023 Table 1 long description.

Survey phase(s)	Question	Response options	Telework measure
3·1	Working from home is sometimes referred to as telework. In the past 7 d, have any adults in this household teleworked?	1) Yes 2) No	Telework = 1 if response = 1 Telework = 0 if response = 2
3·2–3·3	In the past 7 d, have you or your household done any of the following… Teleworked or worked from home?	1) Yes 2) No	Telework = 1 if response = 1 Telework = 0 if response = 2
3·4–3·10	In the last 7 d, have any of the people in your household teleworked or worked from home?	1) Yes, for 1–2 d 2) Yes, for 3–4 d 3) Yes, for 5 or more days 4) No	Telework = 1 if response = 1, 2, or 3 Telework = 0 if response = 4

Additional explanatory variables considered are summarised in Table 2 and include respondent demographics (age, sex, race, ethnicity, educational attainment and marital status), characteristics of the respondent’s household (income as a percentage of the FPL, state of residence, number of adults in household, number of children in household and housing tenure), and survey week.

**Table 2.** Weighted descriptive statistics for low-income US households (< 200 %FPL): HPS, United States, April 2021 to October 2023 Table 2 long description.

	Statistic	Full sample	< 100 % FPL	100–200 % FPL	Pandemic	Post-pandemic
Food insufficiency						
Food-insufficient	%	23·9 %	30·9 %	22·5 %	23·3 %	26·9 %
Food sufficient	%	76·1 %	69·1 %	77·5 %	76·7 %	73·1 %
Telework status						
Telework	%	14·1 %	14·5 %	14·0 %	15·0 %	10·1 %
No telework	%	85·9 %	85·5 %	86·0 %	85·0 %	89·9 %
Income (% FPL)	Mean	142·1 %	81·0 %	154·6 %	143·2 %	137·3 %
HH adults (#)	Mean	2·2	3·3	2·0	2·2	2·2
HH kids (#)	Mean	0·9	2·1	0·6	0·9	0·9
Age (years)	Mean	48·6	42·5	49·8	48·3	49·6
Sex						
Male	%	38·9 %	32·6 %	40·2 %	39·0 %	38·2 %
Female	%	61·1 %	67·4 %	59·8 %	61·0 %	61·8 %
Race/Ethnicity						
White	%	70·5 %	62·7 %	72·1 %	70·7 %	69·6 %
Black	%	18·0 %	22·6 %	17·0 %	17·9 %	18·5 %
Other race	%	11·5 %	14·7 %	10·9 %	11·4 %	11·9 %
Ethnicity						
Hispanic	%	21·4 %	32·0 %	19·2 %	21·4 %	21·4 %
Non-Hispanic	%	78·6 %	68·0 %	80·8 %	78·6 %	78·6 %
Education						
College degree	%	21·4 %	14·9 %	22·7 %	21·4 %	21·3 %
No college degree	%	78·6 %	85·1 %	77·3 %	78·6 %	78·7 %
Marital status						
Married	%	31·4 %	34·7 %	30·7 %	31·1 %	32·4 %
Not married	%	68·6 %	65·3 %	69·3 %	68·9 %	67·6 %
Housing tenure						
Homeowner	%	49·5 %	44·3 %	50·6 %	49·1 %	51·4 %
Non-Homeowner	%	50·5 %	55·7 %	49·4 %	50·9 %	48·6 %
Observations		326 876	46 978	279 898	265 371	61 505

FPL, Federal poverty line; HH, household.

The sample includes households with income below 200 % FPL. Household-level weights were divided by 36 to account for the 36 pooled weeks of data and then applied to account for the HPS’s complex survey design. se was calculated using a Huber-White sandwich variance estimator aggregated at the state level. Full state and week controls were excluded from this table due to space considerations. The post-pandemic period refers to surveys after May 5, 2023.

### Statistical analysis

All analyses were conducted using Stata se Version 18.0. Descriptive statistics were first calculated to characterise study variables. Probit regressions were then estimated to examine the association between telework and household food insufficiency. To account for the complex survey design and the pooling of multiple data collection periods across Phases 3·1 to 3·10, survey weights were adjusted following the US Census Bureau’s technical documentation^(28)^. Because 36 weeks of data were combined to form the study sample, the full-sample household weights were divided by thirty-six to produce an unbiased pooled weight that reflects the target population over the combined study period.

While the US Census Bureau technical documentation outlines a variance estimation framework utilising eighty replicate weights via Successive Difference Replication, probit models can suffer from non-convergence or matrix collapse when analysing highly stratified, specific subpopulations^(28)^. Thus, se was derived using a Huber-White sandwich variance estimator aggregated at the state level. Average marginal effects and baseline predicted probabilities were then estimated using unconditional variance estimation. For telework, the average marginal effect can be interpreted as the predicted average change in the probability of household food insufficiency when a household goes from having no teleworking member to having at least one teleworking member. The predicted probabilities can be interpreted as the average likelihood of a household being food-insufficient, comparing households with no teleworking members to those with at least one teleworking member, holding all other covariates constant at their means.

Sensitivity analyses were run to assess the robustness of model estimates across household income strata and time periods. For household income, separate probit regressions were run for households with income below 100 % FPL and those between 100 % and 200 % FPL. Two time periods were also considered, with probit models estimated for households completing the HPS during the pandemic and households completing the survey post-pandemic (after May 5, 2023).

## Results

The final analytic sample of 326 876 households includes 46 978 households with income < 100 % FPL, of which 7814 reported some telework and 39 164 reported no telework. Among the 279 898 households with income between 100 % and 200 % FPL, 45 934 reported some telework and 233 964 reported no telework. Table 2 presents weighted descriptive statistics for the study sample and for subsamples by household income level and time period. Over 14 % of households in the sample had at least one teleworking member and nearly 24 % reported food insufficiency. Telework prevalence varied with income, with 14·5 % of households with income below 100 % FPL and 14·0 % of households with income between 100 % and 200 % FPL having a teleworking member. Food insufficiency decreased with income, with 30·9 % of households with income below 100 % FPL and 22·5 % of households with income between 100 % and 200 % FPL experiencing food insufficiency. Descriptive statistics also suggested heterogeneity in food insufficiency and telework adoption by time period. Telework adoption was higher during the pandemic, with 15·0 % of pandemic period households reporting a teleworking member compared with 10·1 % during the post-pandemic period. More households reported food insufficiency (26·9 %) during the post-pandemic period compared to during the pandemic (23·3 %).

The primary probit regression estimates are presented in Table 3. Results indicate a significant (*P* < 0·01) inverse association between telework and household food insufficiency for households with income below 200 % of the FPL. The average marginal effect estimate implies a 4·4 percentage point reduction in the probability of household food insufficiency for households with a teleworking member compared to those without. Based on the predicted probabilities shown in Table 4, this represents a 17·1 % decrease in the probability of food insufficiency, decreasing from 24·5 % among non-teleworking households to 20·3 % among teleworking households (*P* < 0·01).

**Table 3.** Weighted probit estimates of the association between telework and household food insufficiency among low-income households (< 200 % FPL): HPS, United States, April 2021 to October 2023 (*n* 326 876) Table 3 long description.

	Coefficient	Average marginal effect
Telework	−0·146**	−0·044**
Income (% FPL)	−0·004**	−0·001**
Adults in household	−0·043**	−0·013**
Kids in household	−0·027**	−0·008**
Age	−0·005**	−0·002**
Female	−0·035**	−0·011**
African American	0·041*	0·012*
White	−0·111**	−0·033**
Hispanic	0·037**	0·011**
College degree	−0·260**	−0·078**
Married	−0·093**	−0·028**
Non-Homeowner	0·247**	0·074**

FPL, Federal poverty line.

* and ** imply significance at *P* < 0·05 and *P* < 0·01 respectively.

Household-level weights were divided by 36 to account for the 36 pooled weeks of data and then applied to account for the HPS’s complex survey design. se was calculated using a Huber-White sandwich variance estimator aggregated at the state level. Average marginal effects were estimated using unconditional variance estimation. The Average marginal effects represent the predicted average change in the probability of food insufficiency when a household has at least one teleworking member compared to none. State and week controls were included in the probit model.

**Table 4.** Weighted probit estimates of the association between telework and household food insufficiency by income and time period:HPS, United States, April 2021 to October 2023 Table 4 long description.

	Full sample	< 100 % FPL	100–200 % FPL	Pandemic	Post-pandemic
Telework					
Coefficient	−0·146*	−0·124*	−0·151*	−0·146*	−0·150*
Average marginal effect	−0·044*	−0·041*	−0·042*	−0·041*	−0·046*
Predicted probabilities					
Telework	0·203*	0·274*	0·189*	0·198*	0·228*
No telework	0·245*	0·315*	0·231*	0·239*	0·274*
Observations	326 876	46 978	279 898	265 371	61 505

FPL, Federal poverty line.

* implies significance at *P* < 0·01 respectively.

The sample includes households with income below 200 % FPL. Household-level weights were divided by 36 to account for the 36 pooled weeks of data and then applied to account for the HPS’s complex survey design. se was calculated using a Huber-White sandwich variance estimator aggregated at the state level. Average marginal effects and baseline predicted probabilities were estimated using unconditional variance estimation. The Average marginal effects represent the predicted average change in the probability of food insufficiency when a household has at least one teleworking member compared to none. Predicted probabilities can be interpreted as the average likelihood of a household being food-insufficient, comparing households with no teleworking members to those with at least one teleworking member. State and week controls were included in the probit models.

Sensitivity analysis results, presented in Table 4, suggest the inverse association between telework and household food insufficiency holds across household income strata. Having a teleworking member (compared to no teleworking member) was significantly (*P* < 0·01) associated with a 4·1 percentage point reduction in the probability of food insufficiency among households with income below 100 % FPL (27·4 % *v*. 31·5 %; *P <* 0·01) and a 4·2 percentage point reduction among households with income between 100 % and 200 % FPL (18·9 % *v*. 23·1 %; *P* < 0·01). This corresponds to a 13·0 % and 18·2 % decrease in the likelihood of food insufficiency among households with income below 100 % FPL and households with income between 100 % and 200 % FPL, respectively.

Results from the sensitivity analysis further indicate the inverse association between telework and food insufficiency is robust across time periods. Having a teleworking household member was significantly (*P* < 0·01) associated with a 4·1 percentage point reduction in the probability of household food insufficiency during the pandemic (19·8 % *v*. 23·9 %; *P* < 0·01) and a 4·6 percentage point reduction during the post-pandemic period (22·8 % *v*. 27·4 %; *P <* 0·01). This indicates telework was associated with a 17·2 % reduction in the probability of food insufficiency during the pandemic period and a 16·8 % decrease in the post-pandemic period.

## Discussion

Results from this analysis indicate a significant (*P* < 0·01) inverse association between telework and household food insufficiency among low-income populations that is robust across income levels (< 100 % FPL and 100–200 % FPL) and time periods (pandemic and post-pandemic). While prior studies establish alternative work arrangements (i.e. part-time employment, variable hours, and working multiple jobs) association with increased food hardship, this study finds telework may have a protective effect^(4,5)^. Specifically, the 4·4 percentage point reduction in food insufficiency for households with a teleworking member suggests the benefits of telework (e.g. increased household income and reduced time constraints) may outweigh its potential drawbacks (e.g. higher household expenses and social isolation) for households with low income^(3)^.

The results of this study have important implications for public health. Food insufficiency poses a significant public health challenge, impacting not only food access but also overall physical and mental health^(8,9)^. Results suggest fostering increased telework adoption, when possible, could be an innovative approach to mitigate food insufficiency and, correspondingly, reduce the incidence of chronic diseases and mental health conditions in the USA. By increasing household income and time availability, telework may encourage adoption of behaviours associated with reduced food insufficiency, including higher food expenditures and greater meal preparation at home^(15–19)^. Future public and private efforts to address food hardship should consider the potential role of alternative work arrangements when developing mitigation strategies.

From a public policy perspective, study findings highlight the potential implications of recent federal telework policy on public health. The Telework Enhancement Act of 2010 required federal agencies to establish telework policies and determine and notify federal employees of their telework eligibility status^(29)^. Findings from this study suggest public policy supporting telework, such as the Telework Enhancement Act of 2010, may promote food sufficiency among participating federal employees’ households. Conversely, a 2025 directive that curtailed routine telework and required most federal employees to return to full-time office work might inadvertently increase household food insufficiency^(30)^. Policymakers should consider including food hardship as an outcome when evaluating the potential impact of public telework policy.

US policymakers might also consider policies to reduce spatial and social inequalities that hinder equitable telework adoption as a means to reduce household food hardship. Factors like limited digital literacy, lack of access to technology and reliable internet, and inadequate home office environments prevent equitable participation in telework^(3)^. While telework shifts work-related costs (equipment, energy, internet) onto employees, current US federal labour laws only mandate reimbursement if these costs reduce earnings below minimum wage^(31)^. Although some states require reimbursement for mandatory remote work, expanding such laws nationwide and including optional telework could improve access^(32)^. Telework access could further be supported by expanding public programmes that increase broadband affordability and support digital literacy education.

### Limitations and future directions

While this study provides important initial insights on the public health implications of telework, future research is needed to empirically investigate the specific mechanisms through which telework affects food insufficiency. The cross-sectional nature of the HPS precludes establishing a causal relationship between telework and household food insufficiency. Potential endogeneity is also a concern, as households with the ability to telework may differ systematically from those who cannot in ways that are unobservable in the HPS but that are correlated with food insufficiency. Future research utilising longitudinal data or a robust instrumental variables approach could address these potential selection biases and establish a more definitive causal link. The HPS also did not provide information on the type of job held by teleworking household members. Future research is needed to analyse the association between telework and food insufficiency in the context of specific occupations and industries. This research would provide an understanding of which industries have the greatest potential for telework among households with low income and provide further insight for public policy development.

Further, while this study highlights the household-level benefits of telework, it is also important to consider the implications of the broader structural adjustments it can cause in the labour market. The literature suggests that widespread telework adoption during the pandemic led teleworkers to leave urban city centres for more affordable regions, contributing to economic instability for local consumer services and the less-mobile workers they employed^(33)^. Research also indicates the direction of the spillover effects of telework in neighbouring communities varies by worker category^(34)^. Future research could examine how these structural adjustments interact with the household-level public health benefits observed in this study to provide a more comprehensive view of telework’s public health impact.

### Public health implications

The findings of this study highlight the potential public health implications of telework. The observed association between telework and reduced food insufficiency suggests broader adoption of flexible work arrangements could contribute to a reduction in food insufficiency. Given that food hardship is a critical public health issue with significant adverse effects on health outcomes and healthcare costs, policies and interventions aimed at promoting telework adoption and equitable telework opportunities may serve as a valuable tool in addressing food hardship and advancing public health goals.

## Table 1. Long description

Table 1 tracks how the primary independent variable, telework, was operationalized despite changes in the HPS instrument over time across Phases 3.1 to 3.10. The table outlines the survey phases, the specific questions asked, the available response options, and how those responses translated to the final binary telework measure. In Phase 3.1, the survey asked if any adults in the household teleworked in the past 7 days, providing simple “Yes” or “No” options, which were coded as 1 and 0 respectively. For Phases 3.2 and 3.3, the wording shifted slightly to ask if the respondent or their household had teleworked or worked from home in the past 7 days, maintaining the binary “Yes” or “No” options and identical 1 and 0 coding. From Phases 3.4 to 3.10, the question inquired if any people in the household teleworked or worked from home in the last 7 days, offering expanded categorical choices of “Yes, for 1-2 days”, “Yes, for 3-4 days”, “Yes, for 5 or more days”, or “No”. To establish a consistent binary measure across the entire dataset, any of the three “Yes” options were coded as a 1, while a “No” response was coded as 0.

Navigate back to Table 1..

## Table 2. Long description

Table 2 details the baseline characteristics of the study’s analytical sample, which includes 326,876 households with income below 200% of the Federal Poverty Line. The data compares the full sample against subsamples stratified by income level, specifically households below 100% FPL (n = 46,978) and those between 100% and 200% FPL (n = 279,898), as well as by time period, distinguishing between pandemic (n = 265,371) and post-pandemic (n = 61,505) observations. Looking at the primary outcome, 23.9% of the full sample experienced food insufficiency, a hardship that was markedly higher for the below 100% FPL group at 30.9% compared to the 100%–200% FPL group at 22.5%, and higher post-pandemic at 26.9% compared to 23.3% during the pandemic. Telework prevalence sat at 14.1% for the full sample, remaining relatively balanced between the lower income tier at 14.5% and the higher tier at 14.0%, but showing a steep decline from 15.0% during the pandemic to 10.1% post-pandemic. Sociodemographic means reveal an overall sample average income of 142.1% of the FPL, an average of 2.2 adults and 0.9 children per household, and a mean respondent age of 48.6 years. Categorical distributions show the full sample is 38.9% male and 61.1% female, 70.5% White, 18.0% Black, 11.5% other races, 21.4% Hispanic, and 78.6% non-Hispanic. Additionally, 21.4% of respondents held a college degree, 31.4% were married, and 49.5% were homeowners. Notes at the bottom explain that household-level weights were divided by 36 to adjust for the 36 pooled survey weeks, standard errors were derived using a Huber-White sandwich variance estimator aggregated at the state level, and fixed controls for state and week were excluded from the table display due to space considerations.

Navigate back to Table 2..

## Table 3. Long description

Table 3 displays the main weighted probit regression model evaluating the association between telework and household food insufficiency among the low-income sample (n = 326,876). Both the underlying probit coefficients and the calculated average marginal effects are provided for every covariate, with asterisks denoting statistical significance. Telework exhibits a highly significant negative association with food insufficiency, with a coefficient of -0.146 and an average marginal effect of -0.044, indicating that having a teleworking household member is associated with a 4.4 percentage point reduction in the probability of food insufficiency (p < 0.01). Significant protective factors that lower the probability of food insufficiency (p < 0.01) include higher income percentage relative to the FPL (AME: -0.001), more adults in the household (AME: -0.013), more children in the household (AME: -0.008), older age (AME: -0.002), female sex (AME: -0.011), White race (AME: -0.033), holding a college degree (AME: -0.078), and being married (AME: -0.028). Conversely, identifying as African American is associated with a significant increase in the probability of food insufficiency with an AME of 0.012 (p < 0.05), as is identifying as Hispanic with an AME of 0.011 (p < 0.01). Non-homeowners show a strong, highly significant positive association with food hardship, carrying a coefficient of 0.247 and an AME of 0.074 (p < 0.01). Technical footnotes confirm that survey weights were scaled by dividing by 36, standard errors were clustered at the state level using a Huber-White sandwich variance estimator, average marginal effects were generated via unconditional variance estimation, and state and week fixed controls were included directly in the background model.

Navigate back to Table 3..

## Table 4. Long description

Table 4 outlines the sensitivity analyses verifying the robustness of the telework-food insufficiency relationship across distinct economic and temporal strata. For the primary telework variable, the probit coefficients remain negative and highly statistically significant (p < 0.01) across all sub-models, recorded at -0.146 for the full sample, -0.124 for households under 100% FPL, -0.151 for households between 100%–200% FPL, -0.146 during the pandemic, and -0.150 post-pandemic. The matching average marginal effects indicate that telework reduces the probability of food insufficiency by 4.4 percentage points in the full sample, 4.1 percentage points for those under 100% FPL, 4.2 percentage points for those between 100%–200% FPL, 4.1 percentage points during the pandemic, and 4.6 percentage points post-pandemic (p < 0.01 for all). The predicted probabilities section demonstrates the baseline likelihood of food insufficiency, showing that when comparing households with telework versus no telework, probabilities drop from 24.5% to 20.3% in the full sample, from 31.5% to 27.4% for households under 100% FPL, from 23.1% to 18.9% for households between 100%–200% FPL, from 23.9% to 19.8% during the pandemic, and from 27.4% to 22.8% post-pandemic. Observation counts confirm the sample sizes for each separate regression model (326,876 full; 46,978 under 100% FPL; 279,898 between 100%–200% FPL; 265,371 pandemic; 61,505 post-pandemic). Technical footnotes reinforce that the models account for scaled weights, state-aggregated standard errors, unconditional variance estimation, and the active inclusion of state and week fixed controls.

Navigate back to Table 4..
